# Tocilizumab (TCZ) may be an effective treatment for poly-juvenile idiopathic arthritis (pJIA) as for rheumatoid arthritis (RA)

**DOI:** 10.1186/1546-0096-10-S1-A55

**Published:** 2012-07-13

**Authors:** Ken-ichi Yamaguchi, Mitsumasa Kishimoto, Masato Okada

**Affiliations:** 1 St Luke's International Hospital, Chuo-ku, Tokyo, Japan; 2 St. Luke's International Hospital, Tokyo, Japan

## Purpose

TNF inhibitors are established treatment for poly-juvenile idiopathic arthritis (pJIA), but there are needs of alternative therapy for refractory patients. Tocilizumab (TCZ) has been widely used for rheumatoid arthritis (RA) in adults, and the effectiveness for systemic JIA has been well-documented. However, the experience on pJIA is limited. We compared the clinical courses of our patients of RA and pJIA to estimate the effectiveness of TCZ.

## Methods

We retrospectively reviewed clinical finding and assessed disease activity index-CRP (DAS-CRP) of four adults with RA and one child with pJIA. Three of four RA are pretreated patients by other biological agents (Infliximab, Adalimumab, Etanercept) and TCZ was first biological agent for one RA and pJIA patients. All patients had significant disease activity (High 4, Moderate 1) when TCZ were administered.

## Results

Three of four RA and a pJIA patients are met the remission criteria of DAS-CRP after 28 weeks administration of TCZ. Another RA patient showed improvement (DAS-CRP 5.19 → 3.65). There was no apparent side effect in any of the patient.

## Conclusion

In this small series, TCZ provided benefit to all of five patients (RA 4, pJIA 1). In the Post Marketing Surveillance of pediatric case of TCZ (132 cases) in Japan, TCZ showed high effectiveness (Remarkable effective 53.0%, Effective 42.4%) and safety in pJIA. TCZ may be a good option to treat not only sJIA but also pJIA.

## Disclosure

Ken-ichi Yamaguchi: None; Mitsumasa Kishimoto: None; Masato Okada: None.

